# Association between brown eye colour in rs12913832:GG individuals and SNPs in *TYR*, *TYRP1*, and *SLC24A4*

**DOI:** 10.1371/journal.pone.0239131

**Published:** 2020-09-11

**Authors:** Olivia S. Meyer, Maja M. B. Lunn, Sara L. Garcia, Anne B. Kjærbye, Niels Morling, Claus Børsting, Jeppe D. Andersen

**Affiliations:** Section of Forensic Genetics, Department of Forensic Medicine, Faculty of Health and Medical Sciences, University of Copenhagen, Copenhagen, Denmark; King Saud University, SAUDI ARABIA

## Abstract

The genotype of a single SNP, rs12913832, is the primary predictor of blue and brown eye colours. The genotypes rs12913832:AA and rs12913832:GA are most often observed in individuals with brown eye colours, whereas rs12913832:GG is most often observed in individuals with blue eye colours. However, approximately 3% of Europeans with the rs12913832:GG genotype have brown eye colours. The purpose of the study presented here was to identify variants that explain brown eye colour formation in individuals with the rs12913832:GG genotype. Genes and regulatory regions surrounding *SLC24A4*, *TYRP1*, *SLC24A5*, *IRF4*, *TYR*, and *SLC45A2*, as well as the upstream region of *OCA2* within the *HERC2* gene were sequenced in a study comprising 40 individuals with the rs12913832:GG genotype. Of these, 24 individuals were considered to have blue eye colours and 16 individuals were considered to have brown eye colours. We identified 211 variants within the *SLC24A4*, *TYRP1*, *IRF4*, and *TYR* target regions associated with eye colour. Based on *in silico* analyses of predicted variant effects we recognized four variants, *TYRP1* rs35866166:C, *TYRP1* rs62538956:C, *SLC24A4* rs1289469:C, and *TYR* rs1126809:G, to be the most promising candidates for explanation of brown eye colour in individuals with the rs12913832:GG genotype. Of the 16 individuals with brown eye colours, 14 individuals had four alleles, whereas the alleles were rare in the blue eyed individuals. rs35866166, rs62538956, and rs1289469 were for the first time found to be associated with pigmentary traits, whilst rs1126809 was previously found to be associated with pigmentary variation. To improve prediction of eye colours we suggest that future eye colour prediction models should include rs35866166, rs62538956, rs1289469, and rs1126809.

## Introduction

The genetics behind human eye colour has been extensively studied. The SNP rs12913832 is strongly associated with eye colour and a good predictor of blue and brown eye colours [1, 2]. rs12913832 is located in the promoter region of *OCA2* and influences the transcription of *OCA2* [3]. The rs12913832:A allele is important for recruitment of transcription factors that positively affect transcription of *OCA2*, and thus production of eumelanin through melanogenesis. In contrast, the rs12913832:G allele has a negative effect on *OCA2* expression and the production of eumelanin [3]. For this reason, individuals with the genotype rs12913832:GG are expected to have blue eyes, while individuals with the genotypes rs12913832:AA or rs12913832:GA are expected to have brown eyes. Multiple genes influence eye colour and several variants associated with normal eye colour variation have been identified through genome wide association studies (GWAS) [4, 5]. However, there are gaps in the knowledge regarding the formation of eye colours. Prediction of eye colours from DNA have applications in forensic genetics. In identification cases and crime cases with no suspect, the prediction of externally visible characteristics, including eye colour, may reduce the number of suspects and allow the police investigators to focus on groups of individuals with the predicted phenotype [6]. Sensitive assays for prediction of eye colour are available. These include the IrisPlex assay, which was developed and validated for use in forensic genetics [7]. Eye colour prediction with the IrisPlex assay heavily relies on the genotype of rs12913832. Hence, prediction accuracies are high for blue and brown eye colours, but low for the so-called intermediate eye colours (green/hazel) [7]. We recently showed that intermediate eye colours are not only genetically, but also phenotypically, poorly understood [8]. It has been suggested that prediction of eye colours should rely solely on the genotype of rs12913832 and be restricted to two categories (blue and brown) [9]. However, the eye colours of approximately 5% Europeans are completely different from what is expected based on the genotype of rs12913832 [2, 10, 11]. Individuals with the genotype rs12913832:GA, who are expected to have brown eye colours, may have blue eye colours, and individuals with the genotype rs12913832:GG, who are expected to have blue eye colours, may have brown eye colours [10, 11]. Hence, although rs12913832 has a large effect on eye colour, other variants with a smaller effect are required for successful eye colour prediction. We previously identified three variants in *OCA2* associated with blue eye colour formation in individuals with the rs12913832:GA genotype [10]. We also examined individuals with the rs12913832:GG genotype and brown eye colour, but did not identify any variants in *OCA2* or in the *OCA2* promoter region with association to brown eye colour. The purpose of the study presented here was to identify new variants in known pigmentary genes, which may explain brown eye colour formation in individuals with the rs12913832:GG genotype. Identification of these variants could help increase the understanding of eye colour biology, but also increase the accuracy for DNA based eye colour prediction. For a summary on melanogenesis we refer to reviews by Kondo and Hearing, and D’Mello et al. [12, 13]. In this study, genes and regulatory regions surrounding the pigmentary genes *SLC24A4*, *TYRP1*, *SLC24A5*, *IRF4*, *TYR*, and *SLC45A2*, as well as the upstream region of *OCA2* within *HERC2* were investigated with massively parallel sequencing (MPS) in 40 rs12913832:GG individuals with different eye colours that were determined by quantitative eye colour measurements.

## Material and methods

### Individuals and ethical compliance

Fourty individuals were selected for sequencing from a database of blood samples comprising 562 Scandinavians (Danes and Swedes) and 217 Italian individuals from two previous studies [9, 11]. All the selected individuals were genotyped as rs12913832:GG in a previous study [9, 11], and all individuals were previously typed with the IrisPlex assay [7, 9, 11]. The use of material (i.e. blood samples) was approved by the Danish Ethical Committee (H-4-2009-125, M-20090237, and H-3-2012-023), the Ethical Committee of Azienda Ospedaliera Ospedal Sant´Anna di Como (U.0026484.23-11-2012), and the Ethical Committee of the University of Milan-Bicocca (P.U. 0033373/12). Participants gave signed consent, and the samples were anonymized. DNA was extracted using the QIAamp DNA Blood Mini Kit following instructions from the manufacturer (Qiagen).

### Quantitative eye colour measurements

Digital photographs of the eyes were taken of each individual as described previously [10, 11]. From the pictures, the ratio of blue pixels vs. brown pixels was determined using the Digital Iris Analysis Tool (DIAT) and the Pixel Index of the Eye score (PIE-score) was calculated [11]. The PIE-score was calculated as: (number of blue pixels − number of brown pixels) / (number of blue pixels + number of brown pixels). Eyes with a PIE-score > 0 had a higher number of blue pixels, and eyes with a PIE-score < 0 had a higher number of brown pixels.

### Probe design and MPS

Capture-probes targeting 604,500 bases in and around *SLC24A4*, *TYRP1*, *OCA2*-*HERC2*, *SLC24A5*, *IRF4*, *TYR*, and *SLC45A2* were designed for the SureSelectXT2 Target Enrichment System using SureDesign software suite (Agilent Technologies) (S1 Table). Samples were prepared for sequencing by following the instructions described in the SureSelectXT2 Target Enrichment System for Illumina Paired-End Multiplexed Sequencing protocol (version F0, 2016) (Agilent Technologies). Paired-end sequencing (2x150bp) was performed on a MiSeq Benchtop Sequencer with a 300 cycle MiSeq Reagent Kit v2 following the manufacturer’s instructions (Illumina) for all analysed regions.

### Analysis of sequencing data

The sequencing output was automatically converted to FASTQ files with the MiSeq Reporter Software. FASTQ-files were trimmed using AdapterRemoval [14] with a minimum read length of 30 bp and Phred quality score of Q = 30, and subsequently aligned to the human reference sequence assembly Feb.2009 GRCh37/hg19 with the Burrows-Wheeler Aligner, BWA-MEM algorithm [15, 16]. Sequence alignment map (SAM) files were converted into binary alignment map (BAM) files using SAMtools [17]. The Genome Analysis Toolkit (GATK) HaplotypeCaller version 4.0.0.0 [18] was used to create Variant Call Format (VCF) files. Variants located in the regions of interest were extracted using BEDTools version 2.27.0 [19]. Genotypes were accepted if the read depth was > 25 and the heterozygote balance (Hb = read depth of allele/read depth of nucleotide position) was 0.15 < Hb < 0.85. Test of associations was carried out in R version 3.5.0 (R Core Team, 2018) using the *fisher*.*test and kruskal*.*test* commands. If less than two individuals were homozygous for the minor allele the genotypes were considered in two groups (homozygous for the major allele and heterozygous or homozygous for the minor allele) and tested with the Wilcoxon Rank Sum test using the *wilcox*.*test* command. Hardy-Weinberg equilibrium (HWE), allele frequencies, haplotypes, and pairwise r^2^ values for linkage disequilibrium (LD) testing were calculated and visualised using Haploview 4.2 [20]. Haploview 4.2 was also used to select tag variants with pairwise r^2^ ≥ 0.8 as threshold. Variants associated with eye colour were analysed with the Ensembl variant effect predictor (VEP) based on data from Ensembl GRCh37 release 94 database [21] and SlideBase [22]. To investigate effects on transcription factor binding sites, sequences of 20 bp encompassing variant loci located in regulatory regions were analysed with PROMO version 3.0.2 [23, 24] using default search settings.

## Results

### Categorisation of eye colours

The eye colours of the selected 40 individuals were categorised into a two category system, blue and brown, based on the PIE-scores [11] (Table 1). A PIE-score of zero reflects a photo with equal numbers of blue and brown pixels [11] in the iris area and, therefore, the eye colour photos with PIE-score > 0 were categorised as blue (n = 24) and eye colour photos with PIE-score < 0 were categorised as brown (n = 16) (Table 1). All 40 individuals were previously typed with the IrisPlex assay [9, 11] and predicted to have blue eye colours (p ≥ 0.85) (Table 1).

**Table 1:** List of samples with PIE-score, eye colour category, IrisPlex prediction, and nationality.

Sample	PIE-score	Eye colour category	IrisPlex prediction (p-value, Blue)	Nationality
1	1.00	Blue	0.96	Danish
2	1.00	Blue	0.96	Danish
3	1.00	Blue	0.91	Swedish
4	1.00	Blue	0.96	Danish
5	1.00	Blue	0.85	Danish
6	1.00	Blue	0.93	Danish
7	1.00	Blue	0.93	Italian
8	1.00	Blue	0.91	Italian
9	0.99	Blue	0.97	Italian
10	0.99	Blue	0.88	Swedish
11	0.98	Blue	0.93	Danish
12	0.90	Blue	0.93	Swedish
13	0.87	Blue	0.91	Italian
14	0.87	Blue	0.96	Danish
15	0.86	Blue	0.88	Italian
16	0.78	Blue	0.93	Danish
17	0.71	Blue	0.91	Swedish
18	0.64	Blue	0.93	Swedish
19	0.55	Blue	0.85	Swedish
20	0.32	Blue	0.85	Swedish
21	0.23	Blue	0.93	Danish
22	0.20	Blue	0.88	Danish
23	0.14	Blue	0.88	Italian
24	0.12	Blue	0.85	Italian
25	-0.01	Brown	0.91	Swedish
26	-0.07	Brown	0.91	Swedish
27	-0.09	Brown	0.85	Italian
28	-0.09	Brown	0.91	Danish
29	-0.17	Brown	0.92	Italian
30	-0.20	Brown	0.91	Danish
31	-0.29	Brown	0.91	Italian
32	-0.36	Brown	0.90	Danish
33	-0.44	Brown	0.85	Swedish
34	-0.50	Brown	0.91	Swedish
35	-0.55	Brown	0.85	Danish
36	-0.62	Brown	0.91	Swedish
37	-0.71	Brown	0.85	Swedish
38	-0.85	Brown	0.93	Swedish
39	-0.89	Brown	0.91	Danish
40	-0.91	Brown	0.85	Italian

### Sequencing of *SLC24A4*, *TYRP1*, *SLC24A5*, *IRF4*, *TYR*, *SLC45A2*, and *OCA2-HERC2*

We sequenced genes and surrounding areas of *SLC24A4*, *TYRP1*, *SLC24A5*, *IRF4*, *TYR*, and *SLC45A2*, as well as the upstream region of *OCA2* within *HERC2* (Table 2 and S1 Table) with the purpose of identifying variants to explain brown eye colours in individuals genotyped as rs12913832:GG. The median coverage across target regions was 136 reads (S1 Table). Positions with coverage less than 25 were not analysed further. A total of 2,216 variants were identified. Twenty-two variants showed significant HWE-departure (*p*-value < 0.05) and were excluded from further analysis. The rs12913832:GG genotype was confirmed in all individuals.

**Table 2:** Regions targeted for Massively Parallel Sequencing (MPS).

Targeted position (hg19)	Target gene	Targeted bases (kb)	Percentage covered (probe design)
chr5:33924931–33986419	*SLC45A2*	50	88%
chr6:380958–421773	*IRF4*	41	95%
chr9:12668608–12710455	*TYRP1*	37	87%
chr11:88547978–89046386	*TYR*	238	78%
chr14:92755906–92967828	*SLC24A4*	177	88%
chr15:28344106–28369332	*OCA2-HERC2*	20	87%
chr15:48394145–48434879	*SLC24A5*	39	95%

Many variants were located in haploblocks (pairwise r^2^ ≥ 0.8), thus in strong or complete linkage disequilibrium (LD). Using the Tagger function in Haploview, we identified 552 independent variants among all target regions. We compared the allele frequencies of the 2,194 variants in the 40 individuals with the categorical eye colour (blue and brown) using Fisher’s exact test. Variants with a raw *p*-value ≤ 0.05 were also tested for association with quantitative eye colour (PIE-score) using the Kruskal-Wallis test. We used the raw *p*-value ≤ 0.05 as inclusion criteria and identified 211 variants in the target regions of *TYRP1*, *SLC24A4*, *IRF4*, and *TYR* with association with eye colour (S2 Table). A total of 65 variants in the *TYRP1* region, 51 variants in the *SLC24A4* region, and 39 variants in the *TYR* region showed associations with both categorical and quantitative eye colours (raw *p*-value ≤ 0.05). Additionally, 45 variants in the *SLC24A4* region and 11 variants in the *IRF4* region showed associations with categorical, but not quantitative eye colour. None of the variants were statistically significantly associated with eye colour when tested under the Bonferroni correction (*p*-value = 0.000091 with m = 552 independent loci) (S2 Table). Of the variants associated with eye colours based on raw *p*-values, we identified several haploblocks (pairwise r^2^ ≥ 0.8) (S3 Table). Interestingly, no variant in the *OCA2-HERC2*, *SLC45A2*, and *SLC24A5* target regions was found to be associated with categorical or quantitative eye colours (*p*-value > 0.05).

### Annotation of variants associated with eye colour

**Fig 1:**
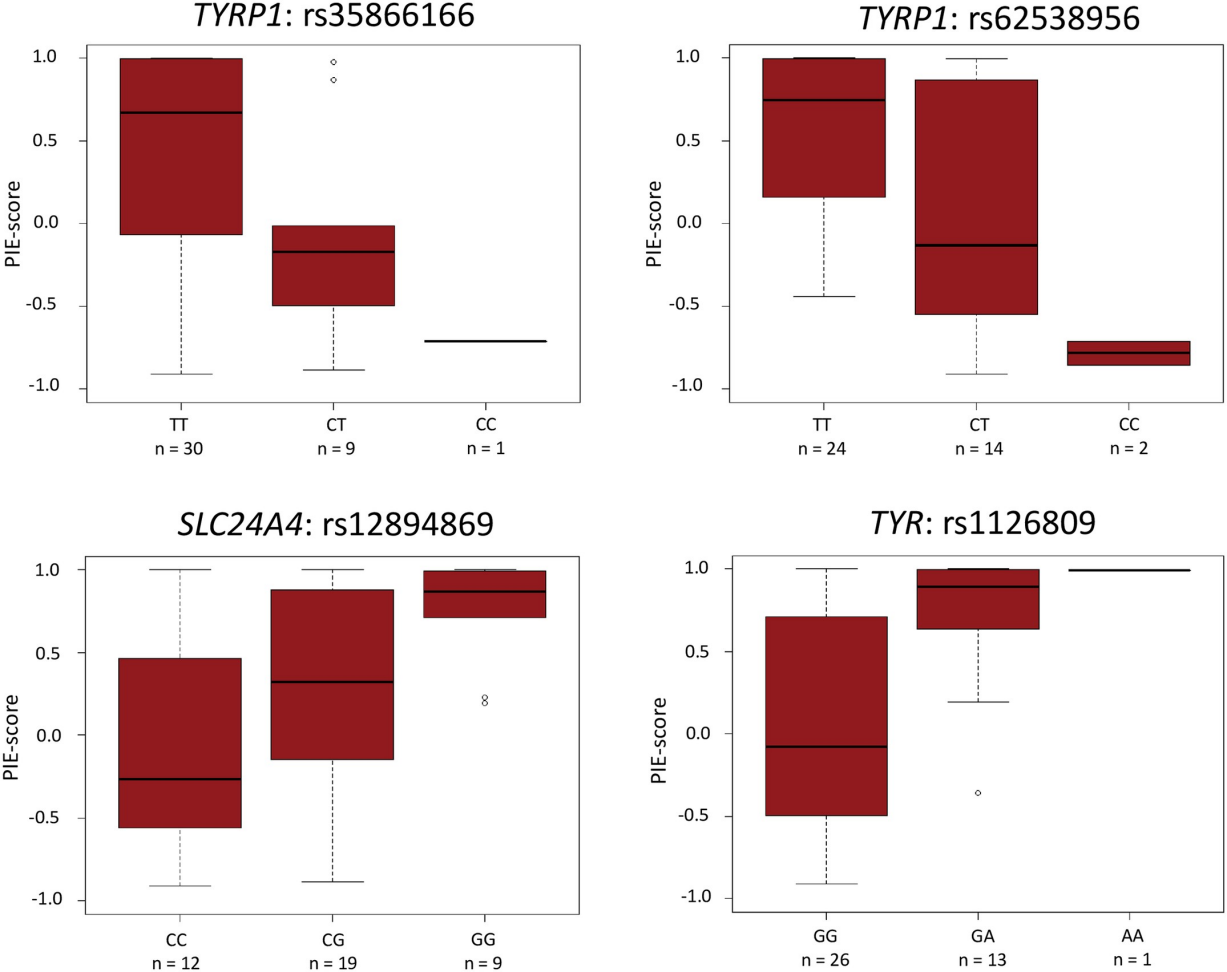
Boxplots showing the distribution of genotypes and PIE-scores. Distribution of genotypes and PIE-scores in 40 individuals with the rs12913832:GG genotype, for the SNPs *TYPR1* rs35866166, *TYPR1* rs62538956, *SLC24A4* rs12894869, and *TYR* rs1126809.

Of the 211 variants, 209 variants were located in intergenic, upstream, or downstream regions of the target genes. One variant (rs7144273) was located within exon 11 of *SLC24A4* and caused a synonymous mutation. rs1126809 was located within exon 4 of *TYR* and caused a missense mutation. A total of 33 variants were located in regions with known or predicted regulatory effects according to annotation by Ensembl variant effect predictor [21] and SlideBase [22]. Furthermore, PROMO [23, 24] was used to assess effects of variants located in regulatory regions (S4 Table). Of the 16 variants in the *TYRP1* target region, one variant, rs62538956, was located in a melanocyte specific enhancer region and predicted to disrupt a binding site for the Yin Yang 1 (YY1) transcription factor. Additionally, three variants were located in enhancers, nine were located in promoter or promoter-flanking regions, and three variants were located within binding sites for the transcriptional repressor CTCF (CTCF binding sites). In the *SLC24A4* region, six variants were located in enhancer regions in intron 10. Five variants were located in CTCF binding sites, and one variant was located in a promoter region. In the *IRF4* region, two variants were located in CTCF binding sites, and one variant was located in a promoter-flanking region upstream of *IRF4*. In the *TYR* region, the variant, rs12273884, was located in an enhancer and predicted to disrupt a binding site for the transcription factor estrogen receptor alpha (ER-α). Finally, rs1126809 in exon 4 of *TYR* caused a missense mutation. Based on the *in silico* predictions, the four most promising variants to explain brown eye colour in rs12913832:GG individuals were *TYRP1* rs35866166:C, *TYRP1* rs62538956:C, *SLC24A4* rs1289469:C, and *TYR* rs1126809:G. Of the 16 individuals categorised with brown eyes, all had at least three of these alleles, and 14 individuals had four alleles. In comparison, only 50% of the individuals categorised with blue eyes had three alleles, and only two individuals had four alleles. The distribution of genotypes and quantitative eye colour (PIE-score) is shown in Fig 1. In rs35866166, rs62538956, and rs1126809, less than two individuals were homozygous for the minor allele. Hence, we carried out additional statistical testing using the Wilcoxon Rank Sum test (raw *p*-values ≤ 0.014). The number of rs35866166:C, rs62538956:C, rs1289469:C, and rs1126809:G alleles found in the 40 individuals, compared with their respective PIE-scores, is visualised in S1 Fig.

### Candidate eye colour SNPs

The Tagger function in Haploview was used to identify the minimal number of tag-SNPs to capture the variation of all 211 variants that showed associations with eye colour in rs129138932:GG individuals. We selected 36 tag-SNPs. Eighteen SNPs in the *SLC24A4* region, eight SNPs in the *TYRP1* region, six SNPs in the *TYR* region, and four SNPs in the *IRF4* region captured 100% of the variants with pairwise LD of r^2^ ≥ 0.8 (Table 3). In the *TYRP1* region, we selected three tag-SNPs to capture 43 variants that were located in the same haploblock and in complete LD (r^2^ = 1). One of the four most promising variants, rs35866166, was part of this haploblock. One tag-SNP in the *TYRP1* target region, rs2762457, was not associated with eye colours, but in strong LD with eight other SNPs associated with eye colours.

**Table 3:** Thirty-six tag-SNPs in the target regions of *IRF4*, *SCL24A4*, *TYR*, and *TYRP1*.

Target gene	Tag SNP^1^	Frequency^2^ Brown (n = 16)	Frequency^2^ Blue (n = 24)	Captured variants (r^2^ ≥ 0.8)
*IRF4*	*rs1050976*	0.66	0.42	*rs56116020*, *rs1050979*, *rs9378805*, *rs1050976*, *rs872071*, *rs9391997*
	*rs12211228*	0.00	0.15	*rs12211228*, *rs6906608*
	*rs10530949*	0.78	0.52	*rs2316515*, *rs10530949*
	*rs9378807*	0.66	0.42	*rs9378807*
*SLC24A4*	*rs10131374*	0.03	0.19	*rs10131374*
	rs11160071	0.53	0.85	rs11160071, rs12435220, rs8008388, rs12883528, rs11623019, rs4904924, rs4904925, rs4904926, rs4904929
	rs12590749	0.09	0.42	rs12590749
	rs12880508	0.47	0.83	rs4904918, rs61977311, rs11160067, rs941648, rs3993878, rs10129711, rs10129728, rs10130041, rs4900124, rs12880508, rs11160069, rs7154848, rs9323877, rs7154400, rs4497611, rs4406992, rs10130373
	rs12894551	0.44	0.75	rs12894551
	rs17128288	0.53	0.19	rs4904896, rs28671668, rs17128288, rs10139051
	rs17128324	0.44	0.10	rs28668079, rs17128324, rs8019291, rs73339573, rs9323876
	*rs34755843*	0.31	0.13	*rs117438089*, *rs34755843*, *rs78487705*, *rs78749625*, *rs61367228*
	*rs35617057*	0.25	0.62	rs10594259, rs8015178, rs12894869, rs12895667, rs7142428, rs11621551, *rs35617057*, *rs4410008*, *rs7143110*, *rs7144261*, *rs12881123*, *rs12882088*, *rs7143416*
	*rs4904887*	0.16	0.37	*rs4904887*
	*rs4904891*	0.53	0.25	rs4243695, *rs4904889*, *rs59778360*, *rs4243696*, *rs4904891*, *rs4904892*, *rs12435024*, *rs10139066*, *rs11624626*, *rs10142146*, *rs10142236*, *rs10142321*, *rs4900120*, *rs11443072*, *rs8010919*, *rs7150295*, *rs4414423*, *rs10143402*, *rs4603491*, *rs10431740*, *rs4904912*, *rs4904913*
	rs4904897	0.38	0.15	rs4904897, rs11624887, rs4904903, rs11160063
	rs4904927	0.66	0.92	rs4904927, rs1998098
	*rs59977926*	0.03	0.31	*rs59977926*
	*rs56007403*	0.23	0.03	*rs56007403*, *rs78863223*, *rs34608909*
	rs7144273	0.37	0.67	rs7144273, *rs7142789*
	*rs7152962*	0.09	0.31	*rs7152962*, *rs2402139*
	*rs7401792*	0.41	0.69	*rs7401792*, *rs12879396*, *rs7155002*
*TYR*	rs2047512	0.09	0.33	rs2047512, rs2648640
	rs7120151	0.94	0.73	rs7120151, rs17184781
	rs34749698	0.06	0.25	rs7118021, rs67279079, rs34749698
	rs11018509	0.06	0.31	rs35702218, rs10765189, rs11018509 rs11018518, rs10830236, rs11018520, rs7949856, rs7925404, rs17791976, rs35486674, rs11018440, rs7103137, rs10830218, rs11018441, rs10830219
	rs1126809	0.03	0.29	rs1393350, rs72963135, rs1126809
	rs9919559	0.16	0.44	rs12273884, rs11018569, rs7947262, rs11018567, rs7924538, rs200640714, rs3907665, rs11018562, rs10830254, rs9919559, rs1827430, rs7951935, rs1806319, rs4121401
*TYRP1*	rs10491745	0.47	0.79	rs10491745, rs10756391
	rs1408799	0.37	0.71	rs1408799, rs1408800
	rs201447946	0.28	0.04	rs75001364, rs77306624, rs112446852, rs76579646, rs74874037, rs202148591, rs77448128, rs75611179, rs77494858, rs79586719, rs78774349, rs80287758, rs111589749, rs76969096, rs112201749, rs77445059, rs117687305, rs74889423, rs11791497, rs11787674, rs11791954, rs112923802, rs76988967, rs62638049, rs61758391, rs61758394, rs11787999, rs34509359, rs141808617, rs74606098, rs77446525, rs139301549, rs35866166, rs112342609, rs77990455, rs149076115, rs79662637, rs201447946, chr9:12703870, rs17280279, rs2209278, rs17280629, rs113819841
	rs74606098			
	rs79586719			
	rs2762457	0.31	0.60	rs2762457, rs10960748, rs10960749, rs13294134, rs10960751, rs10960752, rs13296454, rs59308154, rs2733831
	rs62538950	0.38	0.08	rs62538950, rs16929346, rs59334502, rs16929345
	rs62538956	0.41	0.10	rs144403042, rs62538954, rs62538956, rs62538957, rs62538946, rs79589462

^1^The 36 tag-SNP captured 100% of the variants that showed association with eye colour (r^2^ ≥ 0.8). Variants in italic are associated with categorical, but not quantitative eye colour.

^2^Frequency of variant.

## Discussion

As opposed to GWA studies comprising many individuals and many genetic variants, this study was focused on a highly selected study population comprising few individuals and MPS of selected candidate genes. Given the limited number of individuals included in the study, we combined data from individuals from Northern (Scandinavia) and Southern (Italy) Europe and treated the cohort as one, European population. Although the distributions of Northern and Southern Europeans in each eye colour category were approximately similar (Table 1), we acknowledge that this may have caused bias in the subsequent association analyses. Due to the limited size of the dataset comprising only 40 individuals, our study lacks statistical power. Thus, we fully acknowledge that none of the variants identified in this study showed statistically significant association with eye colours after traditional multiple testing correction. However, we selected a raw *p*-value ≤ 0.05 as threshold for including the variants in further analyses. We are aware that the lenient inclusion criteria cause inclusion of variants that may not be truly associated with eye colours in a larger study population. We also fully acknowledge that due to the size of this study, we are only reporting tendencies of association between SNPs and eye colour. This surely motivates future studies to investigate the associations in larger study populations. In the following section, the most likely causative variants based on *in silico* analyses and revision of the literature are discussed.

We sequenced pigmentary genes of 40 individuals with the rs12913832:GG genotype and identified 211 variants in the *IRF4*, *TYRP1*, *SLC24A4*, and *TYR* target regions associated with brown eye colour. Many of the identified variants were found to be associated with eye colour formation for the first time. It was noteworthy that no variant in *OCA2-HERC2*, *SLC45A2*, and *SLC24A5* was found to be associated with eye colour, even though several variants within these regions have previously been suggested to be associated with pigmentary traits [25, 26]. rs12913832 is located within the sequenced *OCA2-HERC2* region, which is considered one of the most important regions for normal eye colour variation [1, 2]. It was previously shown that no variants in the *OCA2* gene could explain brown eye colour of the tested individuals [10]. In this study, we showed that the variants identified in the *OCA2-HERC2* region comprised one large haploblock in strong LD with rs12913832. Hence, most of the variation within this region was eliminated due to the nature of the dataset. Thus, additional variants in this region could not explain brown eye colour formation in these individuals. The lack of association between eye colour and variants in the *SLC45A2* and *SLC24A5* target regions may also be due to the highly selected dataset. For example, we did not replicate the association between eye colour and *SLC45A2* rs16891982, which is included in the IrisPlex assay [7]. Of the 40 individuals included in this study, one individual with blue eyes (PIE-score = 1) was genotyped rs16891982:GC and the remaining 39 individuals were genotyped rs16891982:GG (S2 Fig). This was expected as rs16891982:G is close to fixation in Europeans (frequency 0.96) and almost completely absent in other populations [27]. Thus, rs16891982 is also a valuable ancestry informative marker (AIM) [28–30] and the association with eye colour may be absent in our study because we only investigated Europeans.

### Variants in the *IRF4* target region

IRF4 has previously been shown to activate the expression of *TYR* through cooperation with the microphthalmia-associated transcription factor (MITF) [31]. One *IRF4* variant, rs12203592, was shown to affect *IRF4* expression in melanocytes [32]. This variant is included in the IrisPlex assay [7]. In this study, we found no association between rs12203592 and eye colours in individuals with the rs12913832:GG genotype (S2 Fig). Variants that were associated with eye colours in the *IRF4* target region were associated with only categorical and not quantitative eye colour. rs12211228 and rs6906608 were in complete LD (r^2^ = 1) (S3 Table). Both are predicted to be located in regulatory regions. rs12211228:C was predicted to disrupt a transcription factor binding site for the tumor suppressor p53 that was previously reported to be linked to skin pigmentation and shown to promote pigmentation in several studies [33, 34]. This hypothesis is supported by the results observed here, where the reference allele, rs12211228:G, with a functional p53 binding site was observed at higher frequency in the individuals categorised with brown eye colour (Table 3). rs6906608 is located in the promoter flanking region of *IRF4*. The Combined Annotation Dependent Depletion (CADD) Phred score for rs6906608 was 13.7 compared to 7.0 for rs12211228, indicating that rs6906608:A is more deleterious than rs12211228:C (S2 Table). However, we did not identify changes in the transcription factor binding site composition as a result of rs6906608:A. Thus, no biological explanation for the association with eye colours was found.

### Variants in the *SLC24A4* target region

*SLC24A4* encodes the calcium transporter SLC24A4. The exact function of SLC24A4 in melanogenesis is unknown. However, SLC24A4 is closely related to another calcium transporter, the solute carrier family 24, member 5 (SLC24A5), which was also sequenced in this study. *SLC24A5* encodes an intracellular potassium dependent sodium/calcium transporter that is highly expressed in melanocytes and involved in the regulation of calcium levels, which is important for melanogenesis [35]. Although the function of SLC24A4 is not known, variants within the *SLC24A4* gene have previously been found to be associated with pigmentation [5, 36]. The SNP rs12896399 is located approximately 15,000 bp upstream of the transcription start site of *SLC24A4* and included as a predictor for eye colour in the IrisPlex assay, where the alternative allele, rs12896399:T, is associated with blue eye colour [7]. In this study, we also observed a higher frequency of rs12896399:T in the individuals categorised with blue eyes (S2 Fig), but the variant did not meet the inclusion criteria. rs17128288, located approximately 100,000 bp upstream of rs12896399 (raw *p*-value ≤ 0.05) is located in a promoter region, and the rs17128288:G allele was predicted to disrupt a transcript factor binding site for the glucocorticoid receptor beta (GRβ). However, the promoter region was not located close to any known transcription start sites. To our knowledge, GRβ has no known function in relation to pigmentation. Hence, despite the high CADD Phred score of 7.2 (S2 Table), we identified no biological explanation for the association between eye colour and rs17128288. Another variant, rs12894869, located within intron 10 of *SLC24A4*, also showed association with eye colours in individuals with the rs12913832:GG genotype. rs12894869 was predicted to be located in an enhancer region. The CADD Phred score for rs12894869 was low (0.6) (S2 Table). The alternative allele, rs12894869:G, was predicted to disrupt two transcription factor binding sites, including a binding site for p53. Similar to rs12211228 in *IRF4*, the reference allele of rs12894869, rs12894869:C, with a functional p53 binding site was observed at a higher frequency in the individuals with brown eye colour. This further supports the hypothesis that p53 may promote pigmentation, and may explain the effect of rs12894869 on eye colour apparent from Fig 1.

### Variants in the *TYRP1* target region

TYRP1 is transferred to melanosomes, where it is involved in the production of eumelanin [37]. Two *TYRP1* variants, rs1408799 and rs683, have previously been associated with eye colour prediction [36]. The importance of rs1408799 was confirmed in this study. Although not located in a regulatory region, rs1408799 showed association with eye colours in individuals with the rs12913832:GG genotype. Two other variants in the *TYRP1* target region, rs62538956 and rs35866166, were predicted to be located in a melanocyte specific enhancer and a melanocyte specific promoter region, respectively. rs62538956 and rs35866166 are located approximately 7 kb and 26 kb, respectively, from rs1408799. The variants were not in LD with rs1408799, or with each other (r^2^ < 0.8) (S3 Table). The alternative allele of rs62538956, rs62538956:C, was predicted to disrupt a YY1 binding site (CADD Phred score = 3.4) (S2 Table). YY1 is a transcription factor that can act as both activator and repressor of gene expression [38]. In 2012, Li and colleagues showed that YY1 regulates expression of genes involved in melanogenesis through cooperation with MITF. The YY1 binding site disrupted by rs62538956:C is located in a predicted enhancer region approximately 14,000 bp upstream of *TYRP1*. Hence, it would be expected that disruption of the YY1 binding site results in decreased *TYRP1* expression. Considering the function of TYRP1 in eumelanin production, reduced expression is expected to result in less pigmentation, and thus blue eye colour. This was not consistent with the results of this study, as rs62538956:C was observed mostly in the individuals with brown eye colours (Table 3). Although we found no explanation of the effect of rs62538956:C, and we were only able conclude on two genotypes and not all the genotypes of the SNP, a clear effect on brown eye colour in rs12913832:GG individuals was seen (Fig 1). It is possible that rs62538956 is in LD with a causative variant, which was not identified or annotated with regulatory effects in this study. The predicted location of rs35866166 was in a melanocyte specific promoter region located directly upstream of a transcription start site of a non-coding *TYRP1* transcript without an open reading frame. It is unknown whether the non-coding *TYRP1* transcript has a role in melanogenesis. However, many non-coding RNAs do have important gene regulating functions and non-coding RNAs have already been shown to influence the pigmentary system in other species [39]. Here, the alternative allele, rs35866166:C was observed more frequently in individuals in the brown eye colour category compared to individuals in the blue eye colour category (Fig 1, Table 3). rs35866166 had a CADD Phred score of 17.6 (S2 Table), and the alternative allele, rs35866166:C, was predicted to generate six additional transcription factor binding sites. It is possible that these sites lead to increased expression of the non-coding *TYRP1* transcript, and that the non-coding *TYRP1* transcript has a positive effect on melanogenesis.

### Variants in the *TYR* target region

In the *TYR* region, the alternative alleles of associated variants were observed at higher frequencies in individuals with blue eye colours compared to the individuals with brown eye colours. Only two of the 39 identified variants, rs7120151 and rs2648640, did not follow this trend, as the alternative alleles were observed at higher frequencies in individuals in the brown eye colour category (Table 3). Both SNPs are located in introns of the *GRM5* gene upstream of *TYR*, but they were not in LD (r^2^ = 0.61) (S3 Table). Neither of the SNPs were predicted to be located in regulatory regions nor to have regulatory effects. The variant rs12273884 was predicted to be located in an enhancer region in intron 3 of *TYR*. The alternative allele rs12273884:C was predicted to disrupt a binding site for the transcription factor ER-α. Estrogen has been shown to positively regulate melanogenesis [40], which corresponds well with the results observed here. We observed a higher frequency of reference alleles, thus a non-disrupted ER-α binding site, in individuals in the brown eye colour category. This could indicate a role for ER-α in the formation of pigment in the eyes. However, whether or not the enhancer element encompassing rs12273884 is in fact an enhancer for *TYR*, and if disruption of the ER-α binding site has an effect on the eye colour formation requires further studies. The only coding variant identified in this study was rs1126809 that is located in exon 4 of *TYR*. The alternative allele, rs1126809:A, introduces a missense mutation that causes the TYR enzyme to be thermosensitive, thus less active [41, 42]. TYR is the rate limiting enzyme in melanogenesis [37, 43]. Hence, individuals with the rs1126809:A allele, and a less active TYR enzyme, are expected to show less pigmentation compared to individuals with the reference allele, rs1126809:G. The results in this study support this expectation. Of the 16 individuals in the brown eye colour category, the frequency of the reference allele was 0.89 (Table 3). In fact, all but one of the individuals in the brown eye colour category were homozygous for the reference, rs1126809:G. The last individual was genotyped heterozygous, rs1126809:GA. rs1126809 has previously been used as a marker for skin pigmentation [5, 44, 45]. Here, we showed that rs1126809 may also influence brown eye colour formation in individuals with the rs12913832:GG genotype (Fig 1). Of all the variants included in this study, rs1126809 had the highest CADD Phred score, i.e. 29.4 (S2 Table). rs1126809 is in LD with rs1393350 (r^2^ = 0.84) (S3 Table), which is included in the IrisPlex assay for eye colour prediction [7]. rs1393350 also showed association with eye colour in this study (S2 Fig, S2 Table). However, rs1393350 had a CADD Phred score of only 1.9 (S2 Table), and does not have a known regulatory effect on pigmentation of the eyes (or skin, or hair) [5]. rs1393350 may still be a good predictor for eye colour. However, we hypothesize that the association between rs1393350 and eye colour is due to LD with rs1126809 and we suggest that rs1126809 rather than rs1393350 are used in future eye colour prediction models.

## Conclusions

In summary, we identified 211 variants in *TYRP1*, *SLC24A4*, *IRF4*, and *TYR*, which may influence brown eye colour formation in individuals with the rs12913832:GG genotype. The variation of all variants was captured by 36 tag-SNPs based on estimated haplotypes (pairwise r^2^ ≥ 0.8). We suggest that the set of tag-SNPs are investigated in a larger study population to examine the association with eye colour further. Due to the limited dataset, use of the raw *p*-values, and the use of *in silico* analyses only, we acknowledge that we may not have identified the true causative variants and all variants associated with brown eye colour in rs12913832:GG individuals. This would require functional studies. Moreover, a substantial increase in the number of individuals included in the study is required to increase statistical power. Nevertheless, based on the raw *p*-values, *in silico* analyses of variant effects, and revision of the literature, we highlighted four variants, rs35866166, rs62538956, rs1289469, and rs1126809, and suggest that these are considered in future eye colour prediction models. Although the functional effects are not completely understood, inclusion of these variants could improve eye colour prediction, even in individuals that do not conform to the expected phenotypes determined by rs12913832.

## Supporting information

S1 Fig Boxplots showing the number of rs35866166:C, rs62538956:C, rs1289469:C, and rs1126809:G alleles in 40 individuals with the rs12913832:GG genotype, compared with their respective PIE-scores. (PDF)

S2 Fig Boxplots showing the distribution of genotypes and PIE-scores for seven SNPs. The distribution of genotypes and PIE-scores for five IrisPlex SNPs: *OCA2* rs1800407, *SLC24A4* rs12896399, *SLC45A2* rs16891982, *TYR* rs1393350, and *IRF4* rs12203592, as well as *GRM5* rs7120151, and *TYR* rs12273884 in 40 individuals with the rs12913832:GG genotype.
(PDF)

S1 Table Positions covered for MPS and coverages of target regions. (XLSX)

S2 Table List of 211 variants of the *IRF4*, *SLC24A4*, *TYRP1*, and *TYR* target regions found to be associated with eye colour in 40 individuals with the rs12913832:GG genotype. *p*-values for Fisher’s exact test (categorical eye colour) and Kruskal-Wallis test (quantitative eye colour), odds ratios (OR) of the alternative alleles calculated for brown vs. blue eye colours (OR > 1 for association with brown eye colour and OR < 1 for association with blue eye colour), minor allele frequency (MAF_EUR) in the European population, and CADD scores are included.
(XLSX)

S3 Table Pairwise r^2^-values (LD) and distances between variants of *IRF4*, *TYR*, *TYRP1*, and *SLC24A4*. (XLSX)

S4 Table Variants in regulatory regions and the predicted effects of the variants. (PDF)
