# Supplementary material for: Association between brown eye colour in rs12913832:GG individuals and SNPs in TYR, TYRP1, and SLC24A4
Source: PLoS One. 2020 Sep 11;15(9):e0239131. doi: 10.1371/journal.pone.0239131 (PMC7485777; doi:10.1371/journal.pone.0239131)
Supplement: S1 Fig — (PDF) [file pone.0239131.s001.pdf]

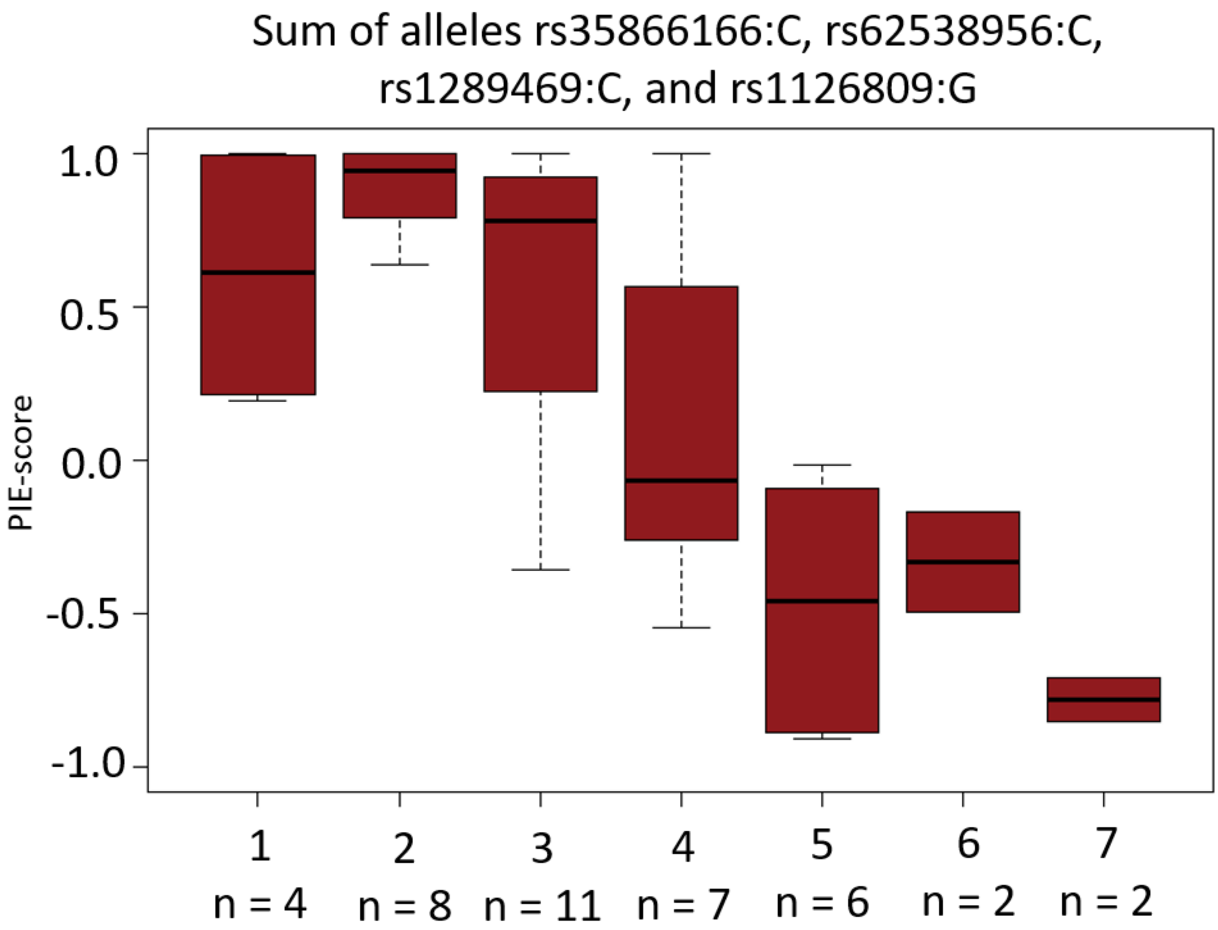

S1 Fig: Boxplots showing the number of rs35866166:C, rs62538956:C, rs1289469:C, and rs1126809:G alleles found in the 40 individuals, compared with their respective PIE-scores.
