# Supplementary material for: Association between brown eye colour in rs12913832:GG individuals and SNPs in TYR, TYRP1, and SLC24A4
Source: PLoS One. 2020 Sep 11;15(9):e0239131. doi: 10.1371/journal.pone.0239131 (PMC7485777; doi:10.1371/journal.pone.0239131)
Supplement: S2 Fig — The distribution of genotypes and PIE-scores for five IrisPlex SNPs: OCA2 rs1800407, SLC24A4 rs12896399, SLC45A2 rs16891982, TYR rs1393350, and IRF4 rs12203592, as well as GRM5 rs7120151, and TYR rs12273884 in 40 individuals with the rs12913832:GG genotype. (PDF) [file pone.0239131.s002.pdf]

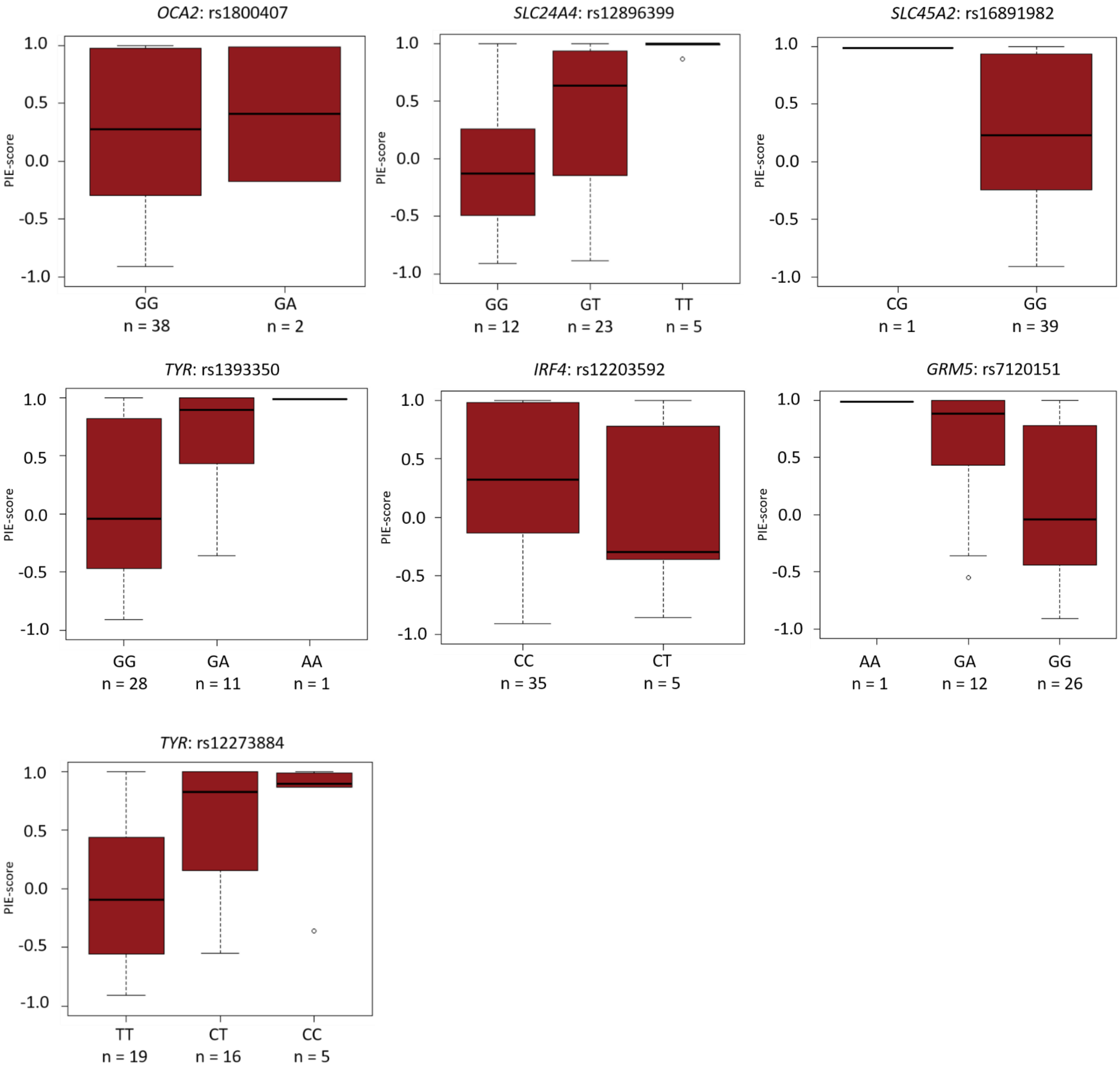

S2 Fig. Boxplots showing the distribution of genotypes and PIE-scores for seven SNPs. The distribution of genotypes and PIE-scores for five IrisPlex SNPs: *OCA2* rs1800407, *SLC24A4* rs12896399, *SLC45A2* rs16891982, *TYR* rs1393350, and *IRF4* rs12203592, as well as *GRM5* rs7120151, and *TYR* rs12273884 in 40 individuals with the rs12913832:GG genotype.
