# Supplementary material for: Association between brown eye colour in rs12913832:GG individuals and SNPs in TYR, TYRP1, and SLC24A4
Source: PLoS One. 2020 Sep 11;15(9):e0239131. doi: 10.1371/journal.pone.0239131 (PMC7485777; doi:10.1371/journal.pone.0239131)
Supplement: S4 Table — (PDF) [file pone.0239131.s006.pdf]

S4 Table. Variants in regulatory regions and the predicted effects of the variants.

Gene	Variant	Genomic location	Variant type	Regulatory region[1]	Effect of variant[1]
TYRP1	rs62538956	12679244	SNP	Melanocyte specific enhancer	1 predicted TFBS is disrupted
	rs62538957	12680607	SNP	Promoter flanking region	3 additional TFBS predicted
	rs79586719	12680486	SNP	Promoter flanking region	1 predicted TFBS is disrupted<br>1 additional TFBS is predicted
	rs78774349	12681428	SNP	Promoter flanking region	1 additional TFBS is predicted
	rs80287758	12684846	SNP	Enhancer	2 additional TFBS are predicted
	rs111589749	12685013	SNP	Enhancer	1 predicted TFBS is disrupted<br>1 additional TFBS is predicted
	rs76969096	12685065	SNP	Enhancer	No change
	rs61758394	12690227	SNP	Promoter flanking region	2 predicted TFBS are disrupted<br>1 additional TFBS is predicted
	rs141808617	12696952	DEL	Promoter	1 predicted TFBS is disrupted
	rs74606098	12697692	SNP	Promoter	1 additional TFBS predicted
	rs77446525	12698003	SNP	Promoter	No change
	rs139301549	12698125	DEL	Promoter	No change
	rs35866166	12698471	SNP	Melanocyte specific promoter	6 additional TFBS are predicted
	rs112342609	12698829	SNP	CTCF binding site	No change
	rs77990455	12699881	SNP	CTCF binding site	No change
	rs149076115	12699921	DEL	CTCF binding site	No change
SLC24A4	rs4904896	92875213	SNP	CTCF binding site	2 predicted TFBS are disrupted<br>1 additional TFBS is predicted
	rs4904897	92875262	SNP	CTCF binding site	2 predicted TFBS are disrupted
	rs17128288	92879794	SNP	Promoter	1 predicted TFBS is disrupted<br>4 additional TFBS are predicted
	rs10139051	92880065	SNP	CTCF binding site	1 predicted TFBS is disrupted<br>1 additional TFBS is predicted
	rs10594259	92916729	DEL	Enhancer	No change
	rs8015178	92916852	SNP	Enhancer	1 additional TFBS is predicted
	rs12894869	92917271	SNP	Enhancer	2 predicted TFBS are disrupted
	rs12895667	92917584	SNP	Enhancer	No change
	rs7142428	92918170	SNP	Enhancer	3 additional TFBS are predicted
	rs11621551	92918741	SNP	Enhancer	1 additional TFBS is predicted
	rs28668079	92927555	SNP	CTCF binding site	2 predicted TFBS are disrupted<br>1 additional TFBS is predicted
	rs17128324	92927591	SNP	CTCF binding site	2 additional TFBS are predicted
IRF4	rs12211228	408833	SNP	CTCF binding site	1 predicted TFBS is disrupted
	rs9391997	409119	SNP	CTCF binding site	No change
	rs6906608	411554	SNP	Promoter flanking region	No change
TYR	rs12273884	89014355	SNP	Enhancer	1 predicted TFBS is disrupted
	rs1126809	89017961	SNP	Missense variant	Reduced catalytic activity of TYR

[1]Regulatory regions and variant effects based on annotation by *Ensembl variant effect predictor*, *SlideBase,* and *PROMO*.
